# Inhibition of Endoplasmic Reticulum Stress Preserves the Integrity of Blood-Spinal Cord Barrier in Diabetic Rats Subjected to Spinal Cord Injury

**DOI:** 10.1038/s41598-017-08052-4

**Published:** 2017-08-09

**Authors:** Zili He, Shuang Zou, Jiayu Yin, Zhengzheng Gao, Yanlong Liu, Yanqing Wu, Huacheng He, Yulong Zhou, Qingqing Wang, Jiawei Li, Fenzan Wu, Hua-Zi Xu, Xiaofeng Jia, Jian Xiao

**Affiliations:** 1 Molecular Pharmacology Research Center, School of Pharmaceutical Sciences, Wenzhou Medical University, Wenzhou, Zhejiang, 325035 China; 2 Department of Orthopaedics Surgery, The Second Affiliated Hospital and Yuying Children’s Hospital of Wenzhou Medical University, Wenzhou, Zhejiang, 325035 China; 3 The Institute of Life Sciences, Wenzhou University, Wenzhou, 325035 China; 4 Department of Neurosurgery, Affiliated Cixi People’s Hospital, Wenzhou Medical University, Ningbo, 315300 China; 5 Department of Neurosurgery, Orthopaedics, Anatomy Neurobiology, University of Maryland School of Medicine, Baltimore, MD 21201 USA; 6 Department of Biomedical Engineering, Anesthesiology & Critical Care Medicine, The Johns Hopkins University School of Medicine, Baltimore, MD 21205 USA

**Keywords:** Experimental models of disease, Spinal cord diseases

## Abstract

The blood-spinal cord barrier (BSCB) plays significance roles in recovery following spinal cord injury (SCI), and diabetes mellitus (DM) impairs endothelial cell function and integrity of BSCS. Endoplasmic reticulum (ER) stress occurs in the early stages of SCI and affects prognosis and cell survival. However, the relationship between ER stress and the integrity of BSCB in diabetic rats after SCI remains unclear. Here we observed that diabetic rats showed increased extravasation of Evans Blue (EB) dye, and loss of endothelial cells and pericytes 1 day after SCI compared to non-diabetic rats. Diabetes was also shown to induce activation of ER stress. Similar effects were observed in human brain microvascular endothelial cells. 4-phenylbutyric acid (4-PBA), an ER stress inhibitor lowered the adverse effect of diabetes on SCI, reduced EB dye extravasation, and limited the loss of endothelial cells and pericytes. Moreover, 4-PBA treatment partially reversed the degradation of tight junction and adherens junction both *in vivo* and *in vitro*. In conclusion, diabetes exacerbates the disruption of BSCB after SCI via inducing ER stress, and inhibition of ER stress by 4-PBA may play a beneficial role on the integrity of BSCB in diabetic SCI rats, leading to improved prognosis.

## Introduction

Spinal cord injury (SCI), a severe trauma, usually leads to large economic burden and permanent disability for patients. Previous research focused largely on improving neurological manifestations through improving sensory function and locomotor function^1, 2^, but the disruption of blood-spinal cord barrier (BSCB) followed SCI still lacks adequate investigation. As a functional equivalent of the blood–brain barrier (BBB), BSCB is composed of a tightly sealed monolayer of endothelial cells and adjacent perivascular cells, which provides physical support and stability for the cellular constituents of the spinal cord^3^. BSCB permeability is primarily determined by junctional complexes, including tight junction (TJ) and adherens junction (AJ), which connect adjacent endothelial cells^4^. Impairment of BSCB may induce spinal cord edema, allow inflammatory cells to enter the injury site, exacerbate secondary injury of SCI, and finally lead to permanent neurological disability^5^.

Diabetes mellitus (DM), characterized by hyperglycemia, is a multi-faceted metabolic syndrome. SCI among DM patients is especially marked by poor outcome, emphasizing the need to understand the pathomechanism of and develop treatment for diabetic patients with SCI. A growing body of evidence suggests that prolonged hyperglycemia facilitates a progressive impairment of neuronal function after SCI in experimental animals and clinical practices^6, 7^. Studies have demonstrated that DM worsens prognosis of SCI by acting through multiple-targets. DM significantly increases oxidative damage^8^, exacerbates neuronal apoptosis^9^ and enhances inflammation^10^. Furthermore, diabetics in rats depresses the expression of PDGF in injured spinal cords, and leads to a slower recovery of motor function^11^. In addition, DM induces a wide range of abnormalities in the cerebral microvasculature^12^. Hyperglycemia induces a direct effect on the integrity of BBB by affecting both TJ and active transport mechanisms^13, 14^, and preclinical studies have revealed that DM is associated with increase of BBB permeability^15, 16^. Only one report showed diabetes decreased occludin expression at the mRNA and protein level and leads to increase of BSCB permeability^17^. Therefore, the exact role of DM in SCI is still unclear, it appears that DM is a risk factor for breaches of BSCB integrity after SCI.

Excessive endoplasmic reticulum (ER) stress appears in injured spinal cord tissue during the acute period following traumatic SCI^18^. When the ER stress is activated after SCI, aggregation of unfolded and misfolded proteins in the ER lumen triggers the unfolded protein response (UPR), which initiates three signaling pathways mediated by PERK, ATF6, and IRE1, subsequently upregulates CHOP expression and caspase-12, and finally leads to cells apoptosis^19^. Except for many studies on the effect of ER stress in neurons, astrocytes, oligodendrocytes, and microglia^20–22^ after SCI, recently, little attention has been paid to the role of ER stress in endothelial cells and BSCB disruption after acute SCI^23^. Fassbender *et al*. reported that mice deficient in CHOP signaling show protective effect in microvasculature after SCI^24^. Our previous studies also showed that, in rat models of SCI, inhibition of ER stress reverses the degeneration of TJ and AJ proteins and protects BSCB integrity^23, 25^. Additionally, GRP78, the ER stress–induced chaperone, is sensitive to glucose concentration, the expression of which was shown to be up-regulated by high glucose (HG) levels in endothelial cells^26^. In past experiments, evidence suggest that ER stress in the endothelial cells might be a potential contributor to vascular complications of diabetes^27^. However, whether inhibiting ER stress is protective for BSCB integrity in diabetes mellitus patients after SCI remains unclear.

Here, we first examined the effects of STZ-induced diabetes on BSCB disruption, ER stress, endothelial cell damage, and pericyte loss after SCI. Previous studies have shown that, in the model of clip-compression injury, therapies administered between 1 h and 5 days (and, most notably, at 24 h post injury) may have an added advantage in reaching their CNS targets^28^. Thus, our study focuses on the change of BSCB structure and function in diabetic rats 1 d after SCI. Rats were administrated with or without 4-phenylbutyric acid (4-PBA), a classical ER stress inhibitor, to better clarify the role of ER stress on BSCB integrity after SCI in diabetic rats. Finally, the pathomechanism of SCI in diabetic rats is further verified *in vitro* via analysis in the co-stimulation of H_2_O_2_ and HG with and without 4-PBA.

## Results

### Successful induction of diabetes with STZ

The level of glucose in blood circulation is the main indicator for the development of diabetic rats^8^. As shown in Supplementary Fig. 1, before STZ injection, there were no significant differences in FPG levels and body weight between diabetes and control groups. From one week to four weeks after STZ injections, FPG levels in all STZ-treated rats were obviously higher than 16.7 mmol/L (a standard glucose level threshold above which is defined as diabetic hyperglycemia), which indicated successful induction of diabetes (Supplementary Fig. 1A, ***p < 0.001). Meanwhile, citrate-treated rats showed normal FPG levels ranging from 1.4 to 6.1 mmol/L. After STZ injections, the body weight of rats in the diabetes group decreased gradually, while the body weights of the citrate-treated rats elevated progressively (Supplementary Fig. 1B, ***p < 0.001).

### DM aggravates disruption of BSCB after SCI

**Figure 1:**
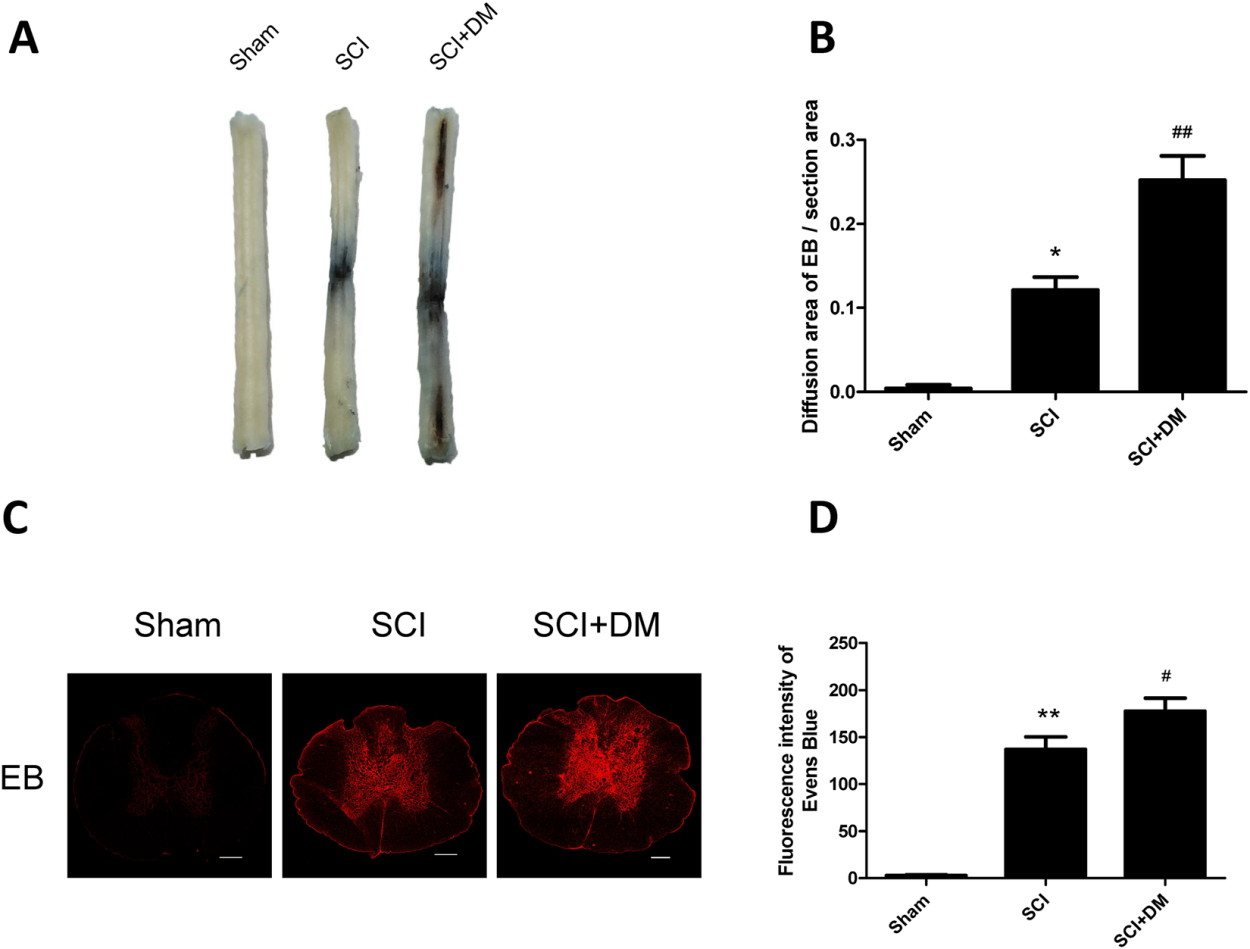
DM aggravates blood–spinal cord barrier (BSCB) permeability after SCI. After SCI, barrier permeability was measured 1d after injury, using Evans Blue (EB) dye in sham, SCI, and SCI + DM group. (**A**,**B**) Representative whole spinal cords and quantification of BSCB permeability data in each group showing EB dye permeabilized into the spinal cord, n = 4. (**C**,**D**) Representative confocal images of EB extravasation and quantification of the fluorescence intensity of EB in each group. Scale bar = 1 mm. n = 4, *p < 0.05, **p < 0.01 vs sham group, ^#^p < 0.05, ^##^p < 0.01 vs SCI group.

To evaluate the effect of diabetes on BSCB integrity, we analyzed the permeability of BSCB 1d after SCI by EB dye extravasation. As shown in Fig. 1A and B, the permeability of EB dye was changed significantly in response to injury as compared with sham control, indicating that injury elicits BSCB permeability (*p < 0.05). However, in SCI + DM rats, the extravasation of EB dye was higher than that in the SCI group 1d after injury (^##^p < 0.01). Similarly, the fluorescence intensity of EB dye was enhanced in the SCI + DM group compared to the SCI group (Fig. 1C and D, ^#^p < 0.05).

Moreover, to evaluate the effect of diabetes on locomotion recovery after SCI, Basso-Beattie-Bresnahan scores and inclined plane test results were evaluated (Supplementary Fig. 2, n = 8 per group). Basso-Beattie-Bresnahan scores were assessed 1, 3, 7, 14, and 28 days after injury. There were no significant differences in the Basso-Beattie-Bresnahan scores between the SCI and SCI + DM groups 1, 3, 7, and 14 days after injury. However, 28d after injury, the Basso-Beattie-Bresnahan score of SCI + DM rats (6.875 ± 0.3981) was lower than that of SCI rats (10.38 ± 0.1830) (Supplementary Fig. 2A, *p < 0.05). Consistent with these findings, the results of the inclined plane test also showed that the maximum angles were markedly lower in SCI + DM rats than that in SCI rats 28d after SCI (Supplementary Fig. 2B, *p < 0.05). All these data suggest that DM further damages locomotor recovery of SCI, which may be partially due to excessive BSCB disruption in diabetic SCI rats.

### DM increases microvessel loss and reduces pericyte coverage after SCI

**Figure 2:**
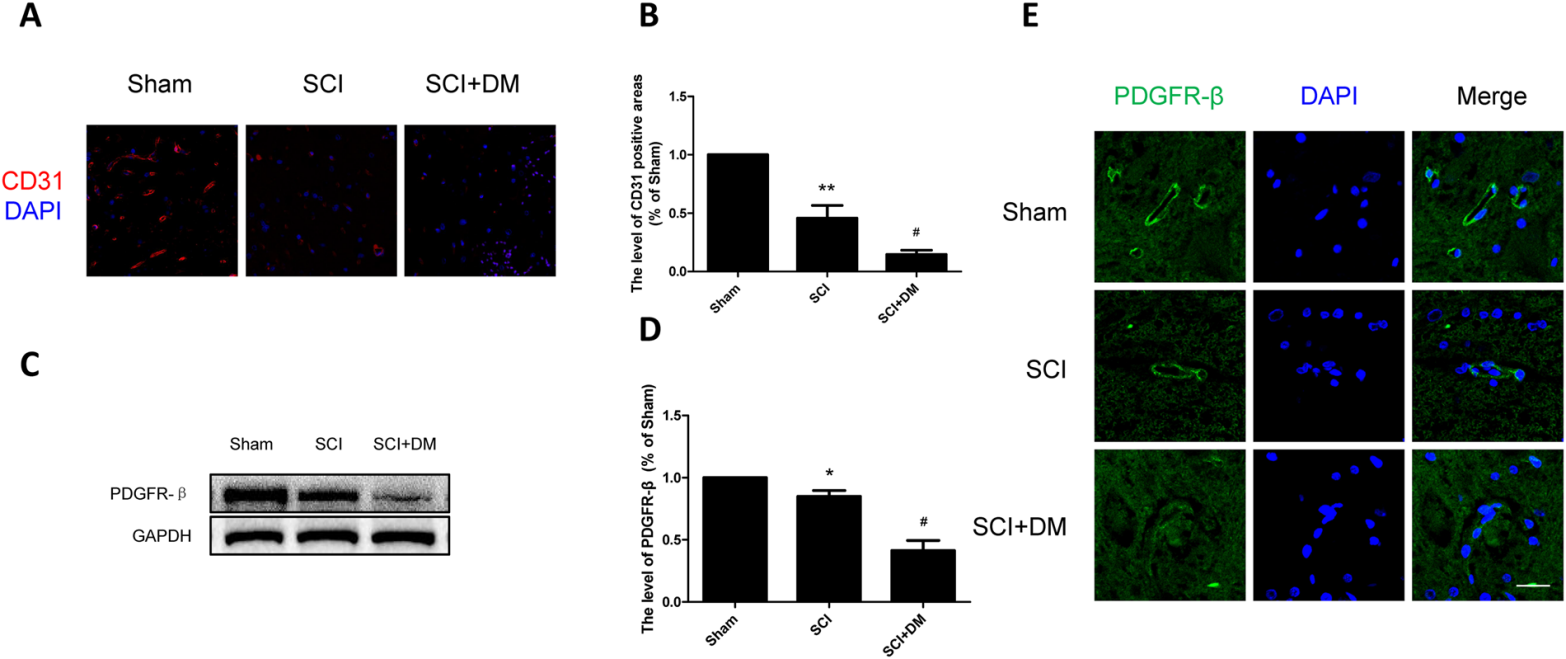
DM aggravates microvessels loss and decreases pericyte coverage after SCI. (**A**,**B**) Immunofluorescence staining of CD31 and quantification of the level of CD31 positive areas in each group, n = 4. Scale bar = 50 μm. (**C**,**D**) Western blot and quantification of PDGFR-β in each group, n = 6. (**E**) Immunofluorescence staining of PDGFR-β in each group, n = 4. Scale bar = 10 μm. *p < 0.05, **p < 0.01 vs sham group, ^#^p < 0.05 vs SCI group.

As an important component of the BSCB, the number and coverage of pericytes determine the permeability of barrier^29, 30^. As shown in Fig. 2A, in the SCI group, less CD 31 (classical endothelial cells marker) positive microvessels were observed in the spinal cord segment 1d after injury than in that of the sham group. Spinal cord segments of the SCI + DM group also showed less CD 31 positive microvessels compared to those of the SCI group (Fig. 2A and B, ^#^p < 0.05). As a specific marker for pericytes, the expression of PDGFR-ß was investigated by western blot and immunofluorescence 1d after injury. Here, we found that the levels of PDGFR -ß were significantly decreased in the spinal cord lesions in the SCI group, with further decrements in the SCI + DM group (Fig. 2C and D, *p < 0.05 vs sham group, ^#^p < 0.05 vs SCI group). Similarly, the fluorescence intensity of PDGFR -β was weaker in the SCI group and further diminished in the SCI + DM group (Fig. 2E), revealing that DM reduces the pericyte coverage.

### DM aggravates the degradation of TJ and AJ proteins after SCI

**Figure 3:**
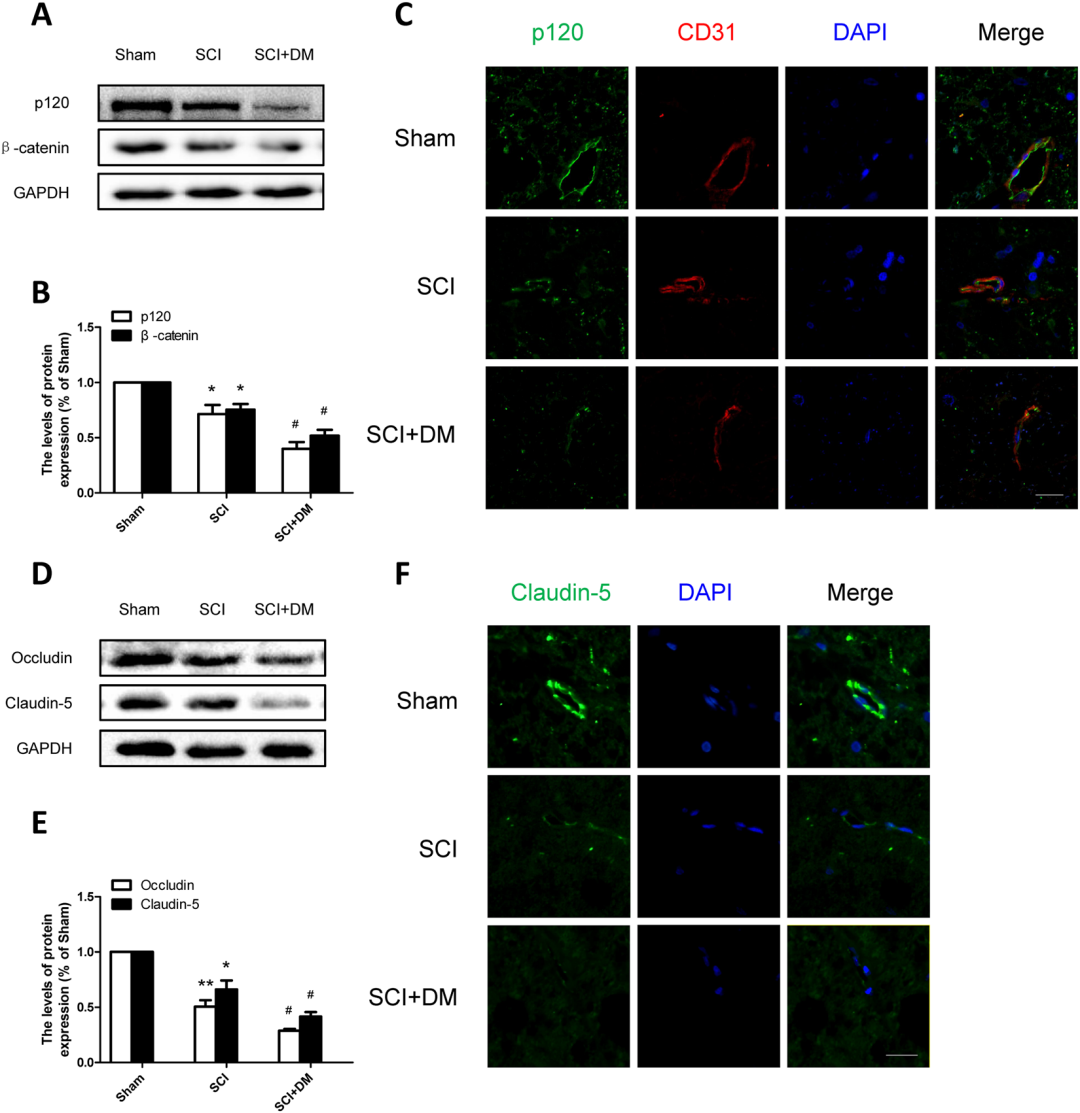
DM aggravates the loss of TJ and AJ proteins after SCI. (**A**,**B**) Western blot and quantification of p120 and β-catenin in each group, n = 6. (**C**) Immunofluorescence staining of p120 and CD31 in each group, n = 4. Scale bar = 10 μm. (**D**,**E**) Western blot and quantification of Occludin and Claudin-5 in each group, n = 6. (**F**) Immunofluorescence staining of Claudin-5 in each group, n = 4. Scale bar = 10 μm., *p < 0.05, **p < 0.01 vs sham group, ^#^p < 0.05 vs SCI group.

TJ and AJ proteins are crucial determinants of the integrity of both BBB and BSCB^4^. It was previously discovered the levels of TJ (claudin-5 and occludin) and AJ (β-catenin, p120) decreased prominently 1d after SCI^4^. As shown in Fig. 3A,B,D and E, the proteins expression of claudin-5, occludin, β-catenin, and p120 were degraded 1d after injury (*p < 0.05, **p < 0.01 vs sham group). Furthermore, the results of western blot revealed that the SCI + DM group showed significantly more decrease in the expression of claudin-5, occludin, β-catenin and p120 1d after SCI compared with the SCI group (Fig. 3A,B,D and E, ^#^p < 0.05). Additionally, the results of immunofluorescent staining showed that the intensity of claudin-5 and β-catenin in the SCI group were decreased after injury compared to the sham group, and the SCI + DM group showed a further exacerbated decrease of intensity (Fig. 3C,F). These data suggest that diabetes aggravates the disruption of BSCB via accelerating the degradation of TJ and AJ proteins after SCI.

### DM exacerbates the ER stress activation after SCI

**Figure 4:**
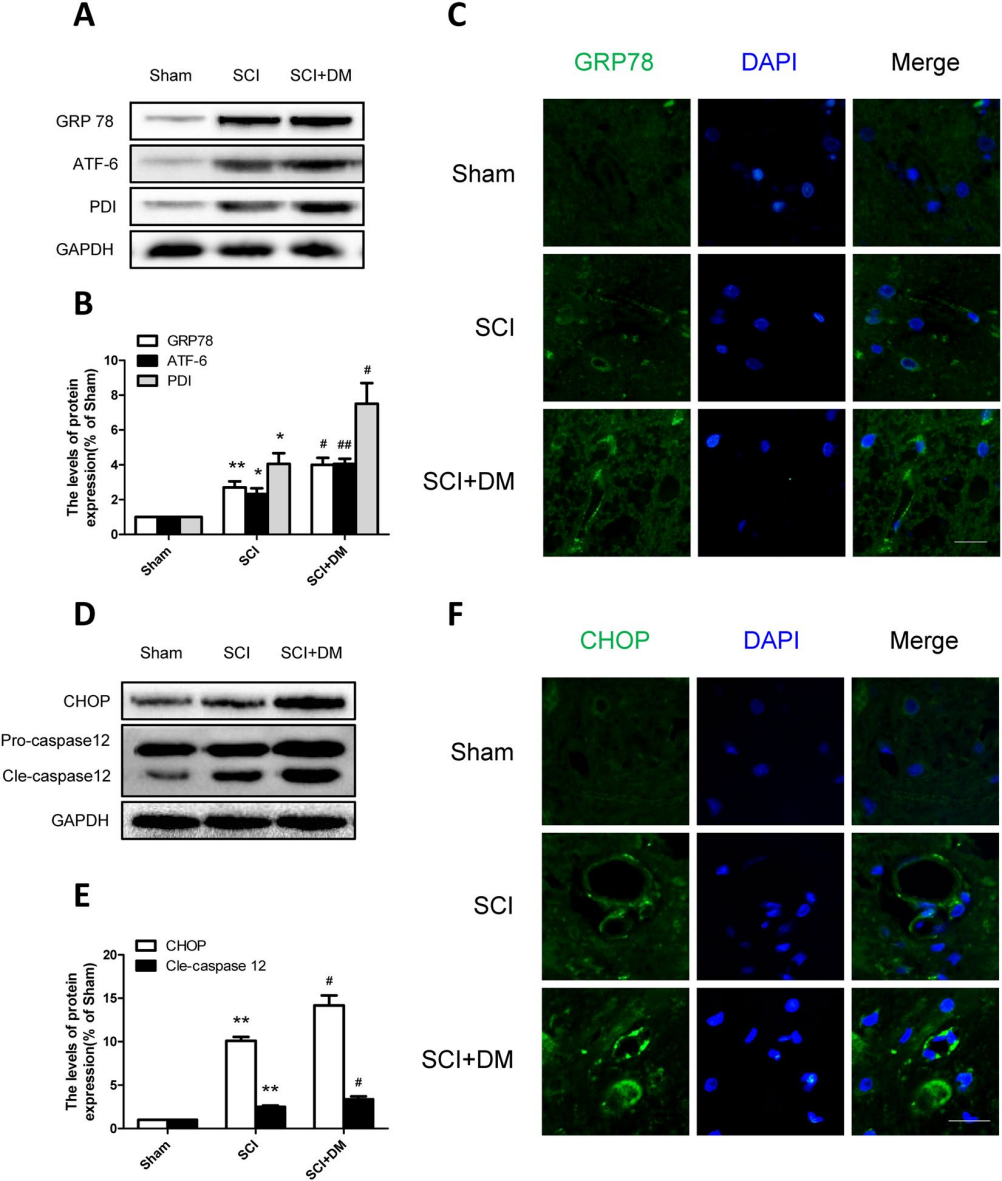
DM exacerbates the activation of ER stress after SCI. (**A**,**B**) Western blot and quantification of GRP78, ATF-6 and PDI in each group, n = 6. (**C**) Immunofluorescence staining of GRP78 in each group, n = 4. Scale bar = 10 μm. (**D**,**E**) Western blot and quantification of CHOP and caspase12 in each group, n = 6. (**G**,**H**) Immunofluorescence staining of CHOP in each group, n = 4. Scale bar = 10 μm. *p < 0.05, **p < 0.01 vs sham group, ^#^p < 0.05, ^##^p < 0.01 vs SCI group.

To verify our hypothesis that diabetes-induced hyperpermeability is related to endoplasmic reticulum (ER) stress, we investigated the levels of ER stress associated proteins including GRP78, ATF-6, PDI, CHOP and caspase-12 by western blot 1d after SCI. As shown in Fig. 4A and B, the levels of GRP78, ATF-6 and PDI were increased 1d after injury in the SCI group and these increases were further exacerbated in the DM + SCI group (*p < 0.05, **p < 0.01 vs sham group, ^#^p < 0.05, ^##^p < 0.01 vs SCI group), which indicated that diabetes further intensifies the activation of ER stress after injury. The SCI group showed that the fluorescence intensity of GRP78 was enhanced in the endothelial cells of spinal cord section after injury compared with the sham group, and the DM + SCI exacerbated the enhancement of its intensity (Fig. 4C). Furthermore, the levels of CHOP and cleaved caspase-12 were markedly increased in the injured spinal cord of the SCI group compared to the sham group, and this increase was significantly aggravated in the DM + SCI group (Fig. 4D and E, **p < 0.01 vs sham group, ^#^p < 0.05 vs SCI group). The immunofluorescence staining results of CHOP were in conformity with western blot results (Fig. 4F). All the data indicate that DM exacerbates the activation of ER stress after SCI, leading to cells apoptosis, which might be the potential reason of diabetes-induced hyperpermeability.

### Inhibiting ER stress attenuates the adverse effects of diabetes in BSCB integrity after SCI

**Figure 5:**
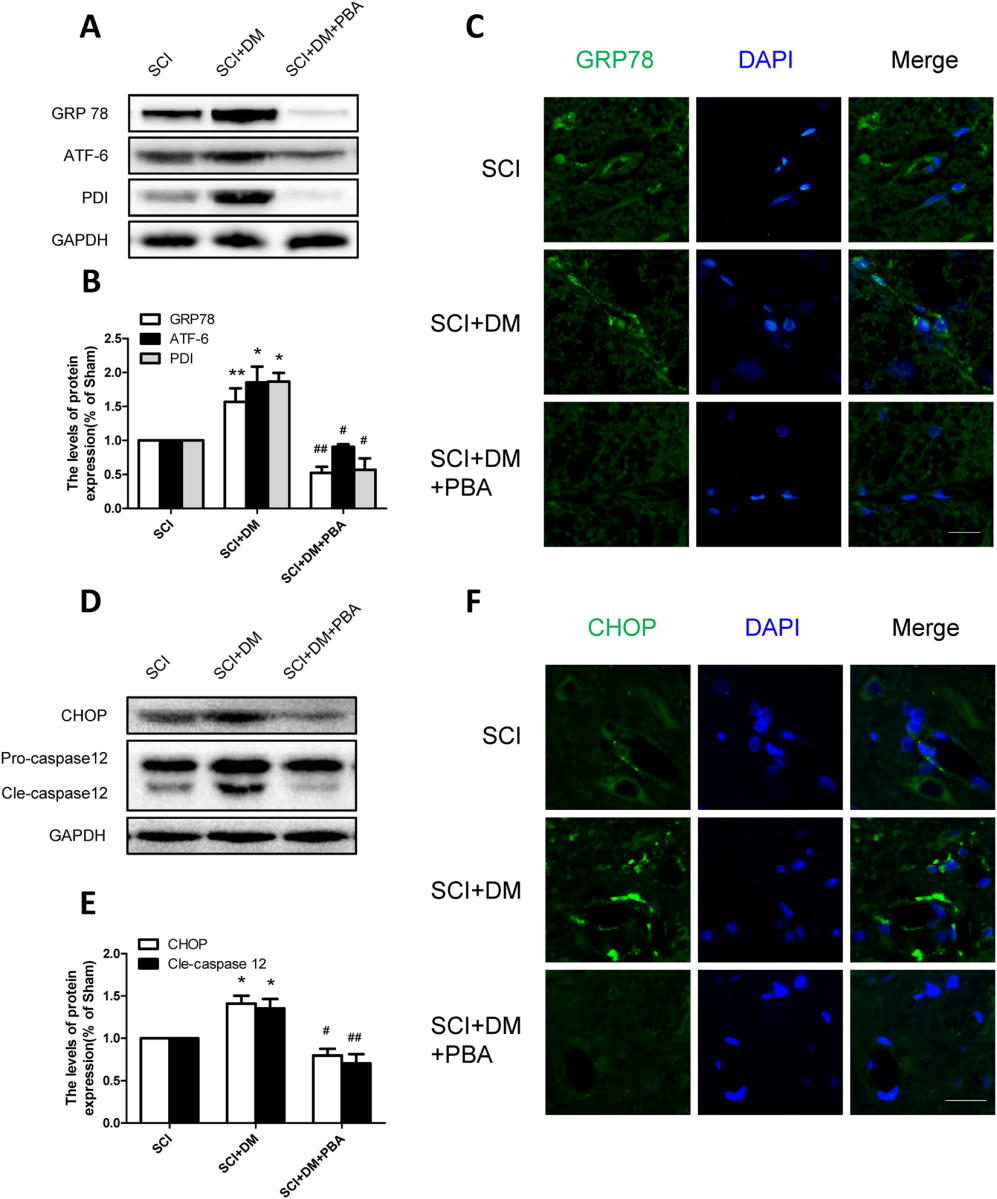
4-PBA preventes the excessive activation of ER stress in diabetic rats after SCI. (**A**,**B**) Western blot and quantification of GRP78, ATF-6 and PDI in each group, n = 6. (**C**) Immunofluorescence staining of GRP78 in each group, n = 4. Scale bar = 10 μm. (**D**,**E**) Western blot and quantification of CHOP and caspase12 in each group, n = 6. (**F**) Immunofluorescence staining of CHOP in each group, n = 4. Scale bar = 10 μm. *p < 0.05, **p < 0.01 vs SCI group, ^#^p < 0.05, ^##^p < 0.01 vs SCI + DM group.

**Figure 6:**
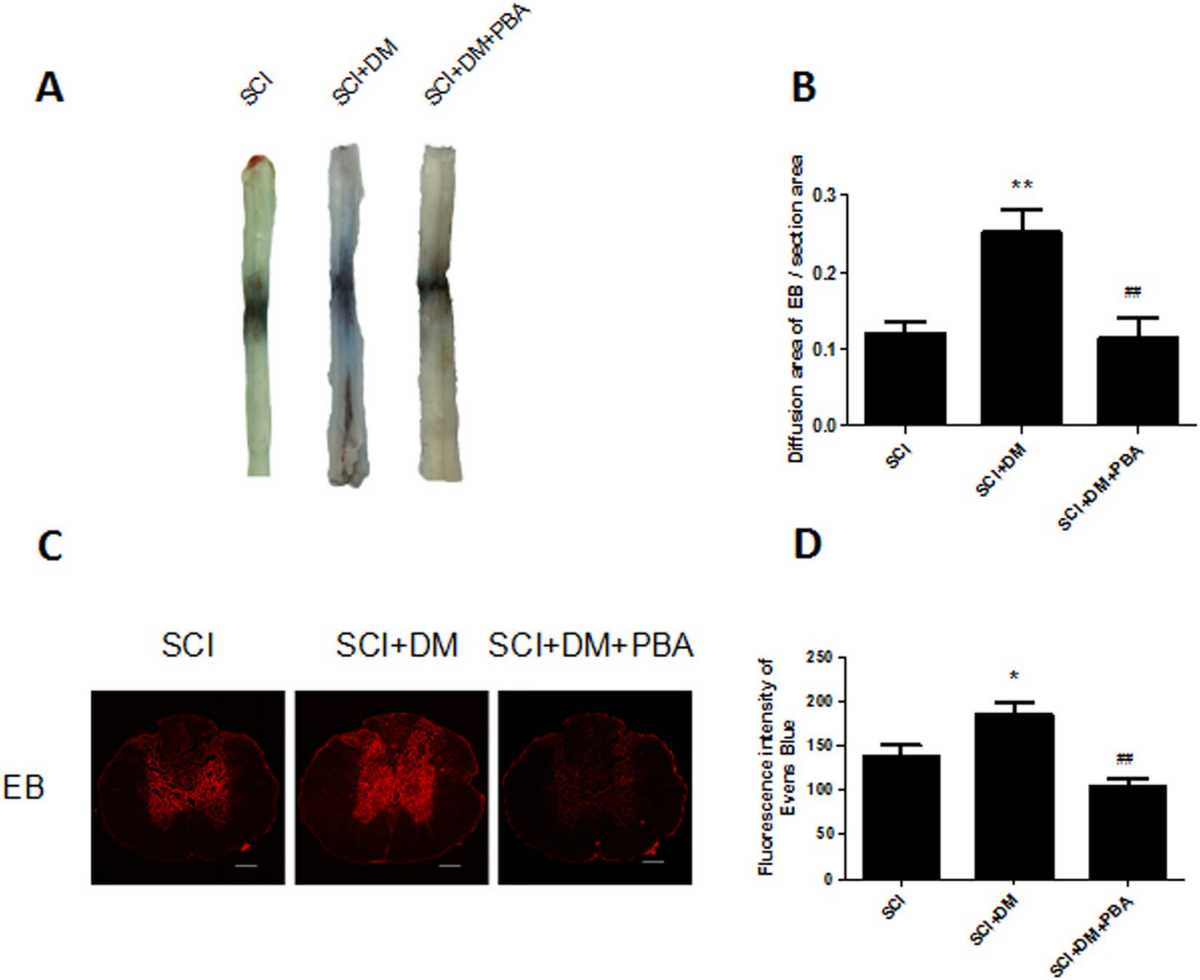
Inhibiting ER stress attenuates the adverse effects of diabetes in BSCB disruption after SCI. After SCI, barrier permeability was measured 1d after injury, using EB dye in SCI, SCI + DM and SCI + DM + PBA group. (**A**,**B**) Representative whole spinal cords and quantification of BSCB permeability data in each group showing EB dye permeabilized into the spinal cord, n = 4. (**C**,**D**) Representative confocal images of EB extravasation and quantification of the fluorescence intensity of EB in each group, n = 4. Scale bar = 1 mm. *p < 0.05, **p < 0.01 vs SCI group, ^##^p < 0.01 vs SCI + DM group.

To verify our hypothesis that excessive activation of ER stress induced by diabetes do harm to the integrity of BSCB after SCI, 4-PBA, an inhibitor of ER stress, was administrated for diabetic rats. According to the results of western blot and immunofluorescence staining, the up-regulation of ER stress associated proteins after injury were reduced by 4-PBA treatment in diabetic rats (Fig. 5, *p < 0.05, **p < 0.01 vs SCI group, ^#^p < 0.05, ^##^p < 0.01 vs SCI + DM group). EB dye extravasation and immunofluorescence of EB dye results revealed remarkable reduction of BCSB permeability in the DM + SCI + PBA group relative to the DM + SCI group (Fig. 6A,B,C and D, ^##^p < 0.01). All the results suggest that the inhibition of ER stress reduces EB dye extravasation, which contributes to maintaining BSCB integrity. 4-PBA treatment also significantly increased the Basso-Beattie-Bresnahan scores 28d after injury (10.75 ± 0.4532) when compared to the DM + SCI group (6.875 ± 0.3981, Supplementary Fig. 3C, ^#^p < 0.05). As shown in Supplementary Fig. 3D, the results of inclined plane test also demonstrated that 4-PBA improved locomotor recovery after SCI (^#^p < 0.05). All the data indicate that 4-PBA reduces and reverses excessive activation of ER stress in diabetic rats after SCI, and is beneficial for locomotor functional recovery and BSCB integrity.

### Inhibiting ER stress prevents microvessels loss and increased pericytes coverage in diabetic rats after SCI

**Figure 7:**
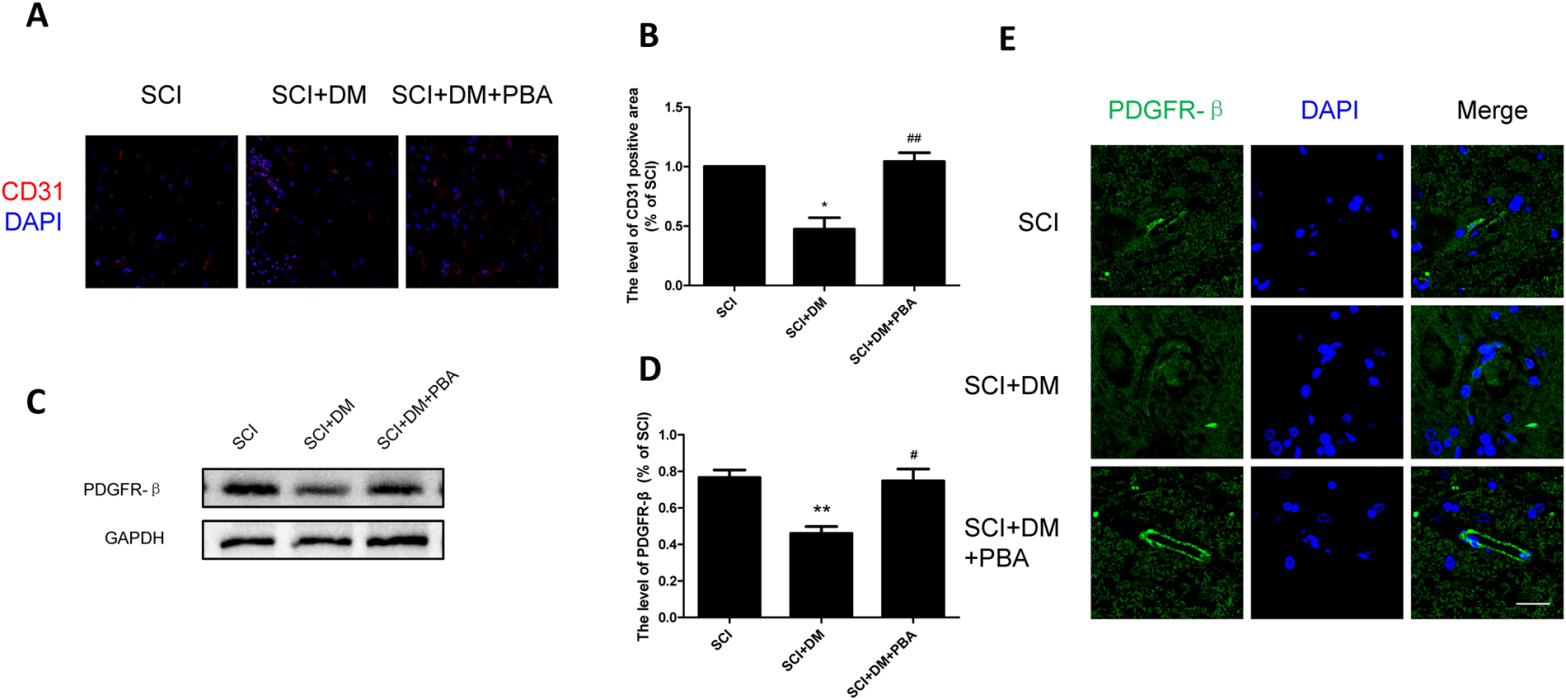
Inhibiting ER stress preventes microvessels loss and increases pericyte coverage in diabetic rats after SCI. (**A**,**B**) Immunofluorescence staining of CD31 and quantification of the level of CD31 positive areas in each group, n = 4. Scale bar = 50 μm. (**C**,**D**) Western blot and quantification of PDGFR-β in each group, n = 6. (**E**) Immunofluorescence staining of PDGFR-β in each group, n = 4. Scale bar = 10 μm. *p < 0.05, **p < 0.01 vs SCI group, ^#^p < 0.05, ^##^p < 0.01 vs SCI + DM group.

Our previous results have shown microvessels and pericytes coverage after SCI were reduced by diabetes due to disruption of BSCB. 4-PBA treatment was shown to markedly increase the protein levels of CD 31 and PDGFR-β (classical pericytes marker) compare to the DM + SCI group (Fig. 7A,B,C and D, ^#^p < 0.05, ^##^p < 0.01). The results of immunofluorescence staining of PDGFR-β were in conformity with the results of western blot (Fig. 7E). All the data suggest that inhibiting ER stress reduces microvessel and pericyte coverage loss, indicating that ER stress inhibitors might play a protective role for BSCB integrity following SCI in diabetic rats.

### Inhibiting ER stress prevents the degradation of TJ and AJ proteins in diabetic rats after SCI

**Figure 8:**
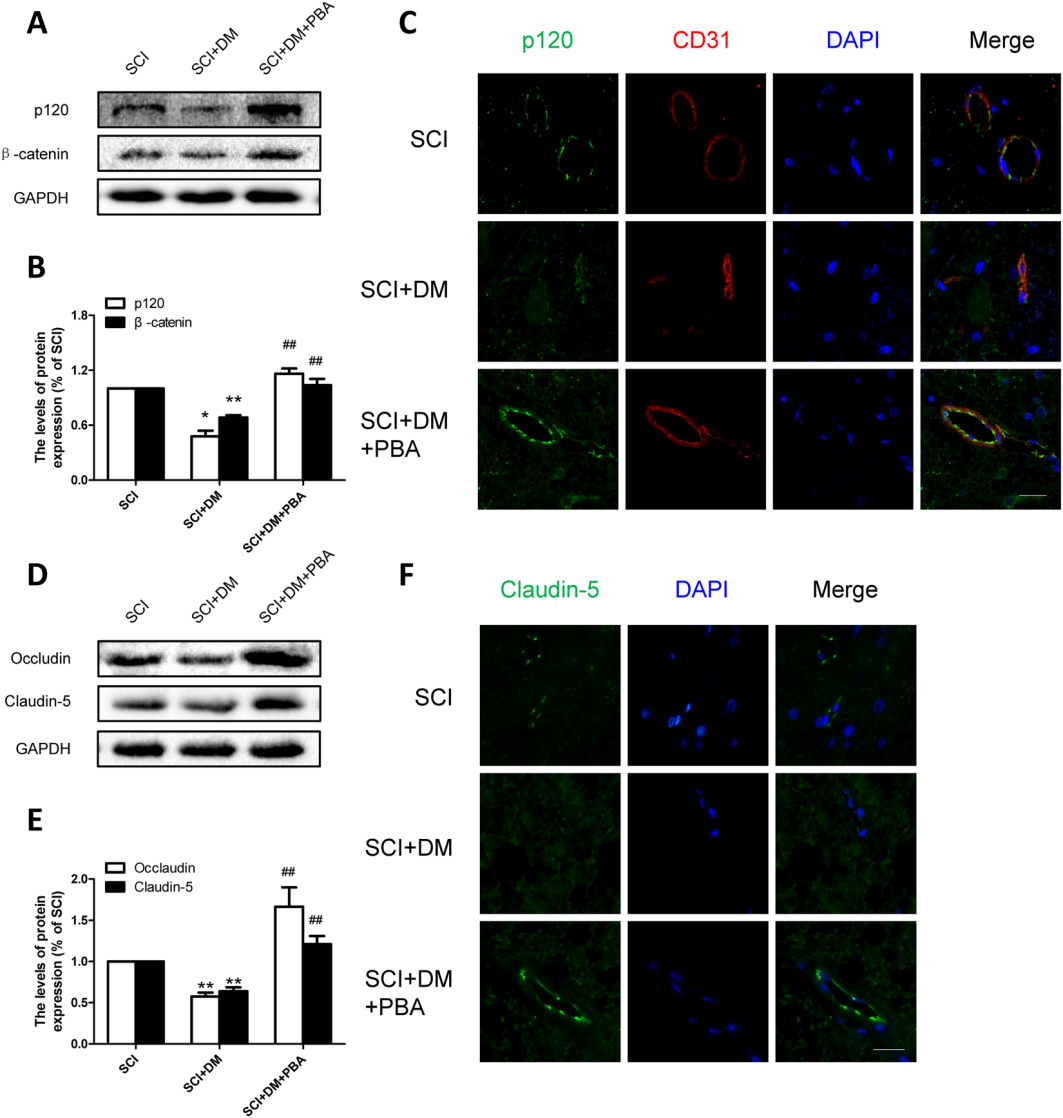
Inhibiting ER stress prevents the loss of TJ and AJ proteins in diabetic rats after SCI. (**A**,**B**) Western blot and quantification of p120 and β-catenin in each group, n = 6. (**C**) Immunofluorescence staining of p120 and CD31 in each group, n = 4. Scale bar = 10 μm. (**D**,**E**) Western blot and quantification of Occludin and Claudin-5 in each group, n = 6. (**F**) Immunofluorescence staining of Claudin-5 in each group, n = 4. Scale bar = 10 μm. *p < 0.05, **p < 0.01 vs SCI group, ^##^p < 0.01 vs SCI + DM group.

The levels of TJ and AJ proteins (claudin-5, occludin, β-catenin, and p120) were evaluated to address the mechanism of ER stress inhibition in diabetic SCI rats. Our results showed that 4-PBA treatment significantly enhanced the levels of claudin-5, occludin, β-catenin, and p120 when compared with the DM + SCI group 1d after SCI by western blot (Fig. 8A,B,D and E, ^##^p < 0.01). Similarly, the results of immunofluorescence of claudin-5 and p120 further confirmed that 4-PBA’s inhibition of ER stress was able to mediate and upregulate junction proteins (Fig. 8C and F). All the results suggest that 4-PBA reverses the degradation of junction proteins in diabetic SCI rats, indicating a potential protective role for inhibitor of ER stress in cases of diabetic SCI.

### Inhibiting ER stress ameliorate HG-induced TJ and AJ proteins loss in H2O2-treated HBMEC

**Figure 9:**
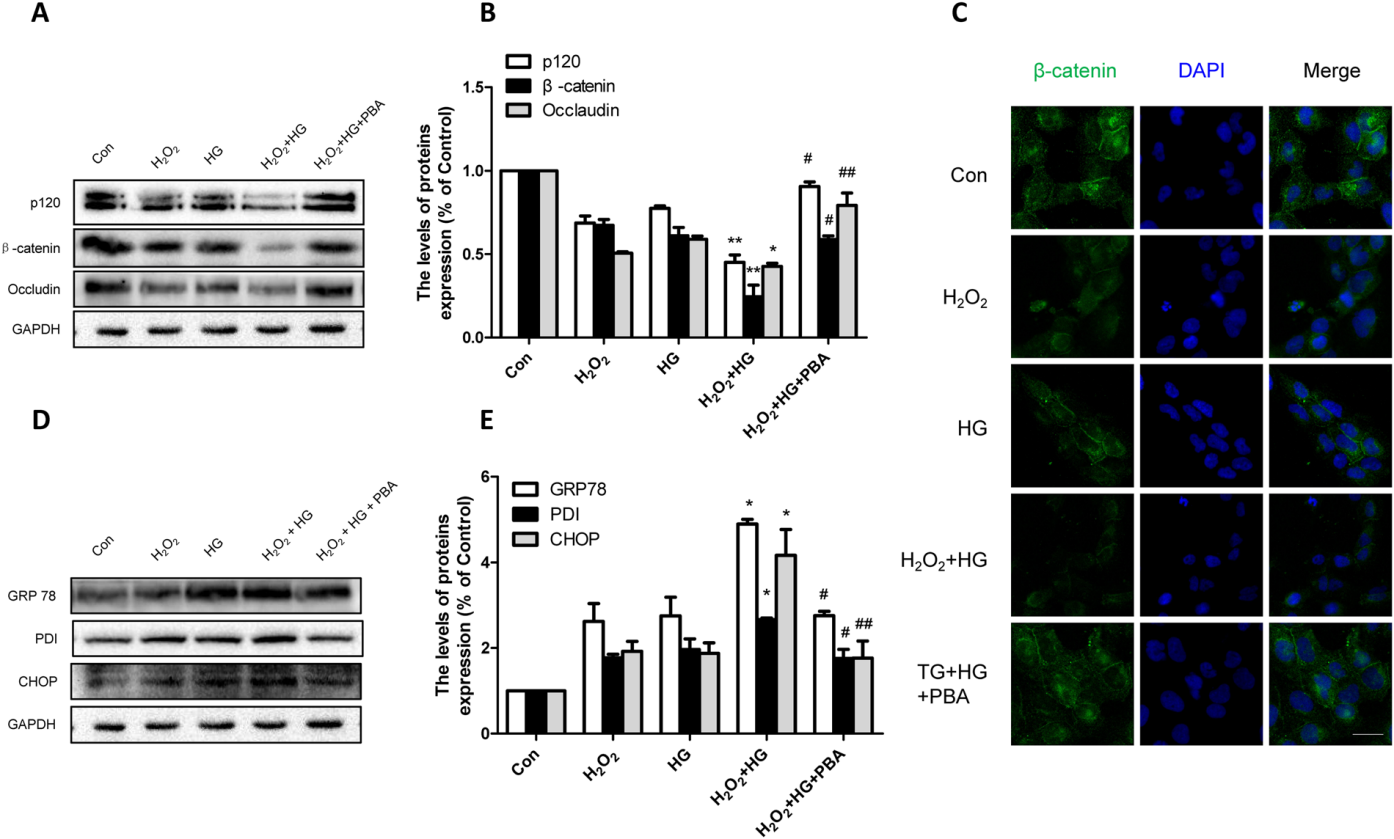
Inhibiting ER stress ameliorates H_2_O_2_ + HG induced loss of TJ and AJ proteins *in vitro*. (**A**–**D**) Western blot and quantification of p120, β-catenin and Occludin in each group. (**E**) Immunofluorescence staining of β-catenin in each group. Scale bar = 10 μm. (**E**,**F**) Western blot and quantification of GRP78, PDI and CHOP in each group. *p < 0.05, **p < 0.01 vs H_2_O_2_ group, ^#^p < 0.05, ^##^p < 0.01 vs H_2_O_2_ + HG group. All experiments were performed three times at least.

To further verify our hypothesis that 4-PBA up-regulates junction proteins via inhibiting ER stress, which contributes to maintaining BSCB integrity, we performed some experiments *in vitro*. To mimic the hyperglycemic and SCI conditions, HBMEC was subjected to HG (20 mg/mL) and H_2_O_2_ (100 μmol/L). As shown in Fig. 9D and E, up-regulation of ER stress associated proteins (GRP78, PDI, and CHOP) in H_2_O_2_ treated HBMEC were seen in the presence of HG concentrations, which was reversed by 4-PBA treatment (*p < 0.05 vs H_2_O_2_ group, ^#^p < 0.05, ^##^p < 0.01 vs H_2_O_2_ + HG group). Consistent with the results *in vivo*, the expression of TJ and AJ proteins were further lowered when compared with that in single H_2_O_2_ treated or single HG treated HBMEC, and this effect was markedly reversed by 4-PBA (Fig. 9A and B, *p < 0.05, **p < 0.01 vs H_2_O_2_ group, ^#^p < 0.05, ^##^p < 0.01 vs H_2_O_2_ + HG group). Similarly, expression of β-catenin detected by immunofluorescence staining further confirmed these results (Fig. 9C). All *in vivo* data once again showed that the protective effect of 4-PBA on maintaining BSCB properties following SCI in diabetic rats is due to the decrease of TJ and AJ proteins loss via inhibiting ER stress.

## Discussion

BBB and BSCB, which are composed of endothelial cells, astrocytes (astrocytic end-foot), pericytes and dense junction proteins, are tight barriers between the circulating blood and central nervous system (CNS). SCI pathology results in rapid, permanent damages to the structure and function of microvessels at the cellular level, leading to increased permeability of BSCB^31, 32^. In our study, we showed, via the evaluation of the extravasation of EB dye, that the BSCB was disrupted and its permeability was significantly increased 1d after SCI (Fig. 1), which agrees with previous reports^33–35^. As a multi-faceted metabolic syndrome, DM has detrimental effects on microvasculature. Prolonged hyperglycemia facilitates the pathophysiology and development of various PNS and CNS disorders^36^. In the co-culture system of HBMEC with human astrocytes, hyperglycemia induced exacerbation of barrier dysfunction was shown to be present as seen by a decrease of transendothelial electrical resistance and increase of EB extravasation^37^. Moreover, Studies have demonstrated that diabetic SCI rats exhibit worse functional recovery, which indicate that diabetic animals will have worse prognoses than non-diabetic animals if the same severity of SCI was inflicted^15^. Consistent with those studies, we found that diabetes inhibits functional recovery after SCI (Supplement Fig. 2). Our results also showed that the extravasation of EB dye following SCI was further increased by diabetes (Fig. 1). Loss of endothelial cells and pericytes, integral components of the BSCB, results in direct microcirculation disruption, and leads to impairment of spinal cord neurons, glial cells and axons^38^. Studies have shown this effect in mice models^39^. Therefore, the expressions of CD31 and PDGFR-β were examined in our study. Our results showed that the endothelial cells and pericytes were lost during injury, and diabetes further enhanced the loss of these two cell types (Fig. 2). The motor function damage immediately following SCI was mainly due to the loss of nerve cells and efferent nerve fibers interruption caused by both primary and second injury. BSCB disruption exposes the spinal cord to the infiltrating inflammatory cells, and high concentrations of amino acid neurotransmitters such as glutamate and glycine, which can be detrimental to neural structures and delay motor function recovery^40^. In accordance with previous studies, in the model of clip-compression injury, the BSCB permeability increased after injury and lasted to day 14 post injury, with most notable changes at 24 h post injury^28^. Our results showed that DM significantly exacerbated BSCB disruption from 6 h to 7 d after injury (Fig. 1 and Supplement Fig. 5). However, the BSCB permeability between SCI and DM + SCI group had not statistical difference at 14 and 28 d after injury (Supplement Fig. 5). It is worth noting that the BSCB change in DM were happening in the early phase of SCI, and the functional decline was only significantly different at day 28 post injury in DM rats than that in vehicle rats (Supplement Fig. 2). Furthermore, DM not only aggravated BSCB integrity, but also exacerbated motor neurons loss after SCI^6, 8^. These two unfavorable factors together delayed motor function recovery. Thus we speculate that the continued BSCB disruption is one risk factor of the behavioral outcomes, which affects the motor function recovery through multiple pathways. Diabetes aggravates BSCB disruption and exacerbated motor neurons loss after SCI, which delays motor function recovery.

It is well known that junctional complexes, important components of the BSCB, comprised of tight, adherens and gap junctions, connect the adjacent endothelial cells, and determined the integrity of BSCB. Any changes in the distribution and expression of junctional proteins are closely related to the permeability of BSCB^41^. Our previous reports show that the levels of TJ and AJ proteins are decreased 1d and 3d after SCI^23^. Diabetes was also reported to impair the permeability of BBB by down-regulating occludin and ZO-1 in STZ-induced diabetes model^13^. In addition, the mRNA and protein levels of occludin were also shown to be decreased following spinal cord injuries in diabetic rats^17^. Our data showed that the levels of occludin and claudin-5 obviously decreased 1d after injury, and hyperglycemia significantly aggravated these reductions, along with those of p120 and β-catenin, both *in vivo* and *in vitro* (Figs 3 and 9). All data suggest that the degradation of AJ and TJ proteins are closely related to the course of disease, and may be potential causes for diabetes-induced increase of BSCB permeability.

The mechanisms by which BSCB breakdown occurs, progresses, and leads to a compromised barrier are manifold^42^. Recently, microvascular ER stress has been confirmed as a potential mechanism for protecting the spinal cord against protracted damage that occurs in the sub-acute phase^24^. Excessive ER stress may lead to apoptosis by activating CHOP and caspase-12, contributing to secondary injury after SCI^43, 44^. Following contusive SCI, CHOP deficiency in mice significantly preserves microvascular density and attenuated macrophage infiltration compared to wild type mice^24^. Up-regulation of GRP78 expression participates in status epilepticus induced vasogenic edema formation and BBB breakdown^45^. Evidence also suggest that ER stress in the endothelial cells may be an important causal factor to diabetes-induced vascular complications^46^, and inhibition of ER stress improves coronary artery function in type 2 diabetic mice (T2DM)^47^. Moreover, our previous studies have also proved that inhibiting ER stress protect BSCB integrity after SCI by attenuating the loss of TJ and AJ proteins^25^. In this study, the expression level of GRP78, ATF-6, PDI, CHOP and caspase-12 were increased 1d after SCI, and this effect was more severe in diabetic rats (Fig. 4). Therefore, we speculate that down-regulation of junction proteins might be ascribed to diabetes-induced excessive ER stress, which contributes to the disruption of BSCB.

4-PBA, an ER stress inhibitor, has been proven to have neuroprotective effects and improve the prognosis of SCI^48^. Furthermore, our previous study showed that 4-PBA prevents disruption of BSCB by inhibiting ER stress after SCI^25^. In addition, 4-PBA is reported to gradually decrease blood glucose in STZ-induced diabetic rats with a dose of 500 mg/kg^49^. Therefore, we utilized 4-PBA as a tool to verify the role of ER stress in BSCB integrity of diabetic rats subjected to SCI. In this study, we focused on the effect of PBA on the BSCB integrity 24 h after SCI. Our results suggest that 4-PBA significantly decreased the BSCB permeability, reversed the upregulation of ER stress associated proteins (Fig. 5), and promoted functional recovery compared with DM + SCI group (Supplement Fig. 3), which means that the inhibition of ER stress contributes to functional recovery in diabetic rats after SCI. However, we also found that 4-PBA did not reduce the glucose level (Supplement Fig. 3A), which may be attributed to the administration dosage and suffering from SCI. Moreover, the precise role of 4-PBA in regulation blood glucose remains controversial in diabetic rats^49^. Interestingly, in diabetic rats after SCI, 4-PBA increased the levels of TJ and AJ proteins (Fig. 8), reduced the loss of endothelial cells and pericytes (Fig. 7), and finally reduced the disruption of BSCB according to the evaluation of the extravasation of EB dye compared to the DM + SCI group (Fig. 6). Therefore, we conclude that diabetes may complicate SCI by inducing an increase in the ER stress response, which subsequently results stronger BSCB disruption and worse prognosis, and all of which can be reversed by 4-PBA treatment via inhibiting of ER stress. *In vitro*, H_2_O_2_ and HG were used to partly mimic SCI and diabetes respectively. Consistent with our results *in vivo*, the reduction of AJ and TJ proteins in the co-stimulation of H_2_O_2_ and HG were reversed by 4-PBA administration in HBMEC (Fig. 9). The current findings suggest that ER stress plays a significant role in BSCB disruption in diabetic SCI rats, and that the regulation of ER stress related pathways might provide a new target for preventing BSCB disruption in diabetic SCI rats.

As with all research, this work was not without limitations. One of the most significant limitations in this research is that the STZ-induced model of diabetes is more reflective of T1DM, and therefore does not accurately reflect all aspects of the more common T2DM. In our research, we first investigated DM’s effect on BSCB disruption after SCI. In the model of clip-compression injury, the BSCB is disrupted as early as 1 h after injury, with maximum disruption24h after injury^28^. Our study only focused on the change of BSCB structure and function in diabetic rats at 24 h after SCI, and did not further explore the change of BSCB permeability, vessel and pericyte loss, junction proteins downregulation, or ERS associated proteins expression at longer time after injury. Other reports have shown that both CHOP and caspase 12 were higher in in diabetic rats 7 d after SCI^7^. Thus, we speculated the damage of BSCB integrity was drastically aggravated 24 h after injury and lasted longer in diabetic SCI rats, which is worth further investigation. Results of this study suggest that the protection of 4-PBA in BSCB integrity was due to inhibition of ER stress. To further confirm the precise mechanisms underlying the 4-PBA protecting BSCB integrity, CHOP knockout mice should be employed in future investigations.

Inflammation, oxidative stress, autophagy *et al*. also play important roles in the secondary injury of SCI^50^. Previous studies have found that increased inflammatory response^10^, oxidative stress^9^ and autophagy^6^ in rats with hyperglycemic condition and SCI. However, the potential mechanisms of DM on the structure and function of BSCB following SCI remain unclear. Further studies are warranted to investigate the potential mechanisms of DM on BSCB disruption after injury to enhance the recovery following SCI in normal and diabetic subjects.

In conclusion, our study showed that STZ-induced diabetes significantly exacerbated BSCB disruption after SCI in rats through loss of endothelial cells and pericytes, and also through degradation of TJ and AJ proteins. While induction of ER stress was found to be associated with more dramatic BSCB disruption after SCI in diabetic rats, and inhibition of ER stress by 4-PBA protected BSCB integrity via reducing the degradation of TJ and AJ proteins. Considering the prevalence of diabetes among the world population, inhibitors of ER stress represent a promising new therapeutic strategy for protecting BSCB integrity after SCI for DM patients.

## Materials and Methods

### Animals

All animal experiments were approved by the ethics committee of Wenzhou Medical University. All procedures were carried out in accordance with the Guide for the Care and Use of Laboratory Animals. Adult female Sprague-Dawley (SD) rats (220–250 g) were obtained from Animal Center of Chinese Academy of Science and maintained in a temperature controlled environment (23–25 °C) with 12 h light/dark cycles and free access to food and tap water for at least 7 d before the experiments.

### Animal model of type 1 diabetes mellitus (T1DM)

All rats (n = 100) were randomly divided into two groups: (1) control group, and (2) diabetes group. 1% STZ was purchased from Sigma (Sigma–Aldrich, St. Louis, MO, USA), which was dissolved in citrate buffer. After 14 h of food deprivation, the rats in the diabetes group were intraperitoneally (i.p.) injected with 1% STZ (60 mg/kg), and the rats in the control group were treated with a vehicle citrate buffer (1 ml/kg) instead. Establishment of T1DM in the diabetes group was confirmed 1 week after STZ injection by measuring fasting plasma glucose (FPG) levels (>16.7 mmol/L) with an autoanalyser (Sinocare, Changsha, Hunan, China). FPG levels and body weight were measured for all animals both before injection, as well as 1, 2, 3, and 4 weeks after injection.

### Animal model of SCI

Within the control group (n = 40), rats were randomly divided into either the sham control or SCI groups. Meanwhile, diabetic rats (n = 51) were randomly divided into the DM + SCI and DM + SCI + PBA groups. Rats in the SCI, DM + SCI and DM + SCI + PBA groups were subjected to SCI 4 weeks after STZ injection. Rats were i.p. injected with 2% pentobarbital sodium (40 mg/kg, Solarbio Science & Technology, Beijing, China) for anesthesia. After the T9 vertebrae of rats were located, all fur and muscle adjacent to the spinous processes were dislodged to expose the vertebral column, and a laminectomy was operated at the T9 vertebral level to expose the spinal cord. Then, crush injury was inflicted via compression using a vascular clip (15 g forces, Oscar, Suzhou, Jiangsu, China) for 2 min. The incision sites were then closed in layers and a topical antibiotic (cefazolin sodium salt, 50 mg/kg, i.p.) was applied. 100 mg/mL 4-PBA was purchased from Sigma (Sigma–Aldrich, St. Louis, MO, USA), which was dissolved in 100% DMSO and diluted with anhydrous alcohol. After injury, the rats in DM + SCI + PBA group were immediately i.p. injected with 4-PBA (100 mg/kg), and repeated once a day for 4 weeks. Equal doses of DMSO were administered for vehicle control. Rats in the sham control group were subjected to the same surgical procedure without a compression injury. After injury, manual urinary bladder emptying was performed twice daily until bladder function recovered. Following completion of the trial, rats were euthanized via pentobarbital sodium overdose respectly 1d and 28d after SCI.

### Locomotion Recovery Assessment

Functional deficit examinations were performed using Basso-Beattie-Bresnahan locomotion scale and inclined plane test according to the previous reports^51^. These tests were performed by blinded independent investigators 1, 3, 7, 14 and 28 days after SCI. Crawling ability was evaluated with the Basso-Beattie-Bresnahan scale, and scores ranging from 0 (no observed hindlimb movements) to 21 (normal locomotion) were recorded. The inclined plane test was also performed on a testing apparatus (a board covered with a rubber mat containing horizontal ridges spaced 3 mm apart). All the rats were tested in two positions (right-side up or left-side up) on a testing apparatus. For each position, the maximum angle at which a rat could retain its position for 5 s without falling was recorded, and these values were averaged to obtain a single score for each rat.

### Measurement of BSCB Disruption by Evans Blue (EB) dye

The integrity of the BSCB can be examined with EB dye extravasation as described elsewhere^52^. 1 day after SCI, rats were injected via tail vein with 2% EB (4 ml/kg, Sigma–Aldrich, St. Louis, MO, USA), and euthanized with overdose of pentobarbital sodium two hours later. Then, the rats were transcardially perfused with 0.9% NaCl, followed by 4% paraformaldehyde. The spinal cord segments near the lesion epicenter were separated into 20-μm thick sections with a cryostat. The fluorescence of EB in spinal tissues was observed by a confocal microscopy and the relative fluorescence intensity was quantified using Image ProPlus (Media Cybernetics, Rockville, MD, USA).

### Immunofluorescence

16 rats were euthanized with pentobarbital sodium overdose, then transcardially perfused with 0.9% NaCl followed by 4% paraformaldehyde 1d after injury. The spinal cord segments near the lesion epicenter were fixed in 4% paraformaldehyde for 24 h and embedded in paraffin. Then, 5 μm-thick thick sections were cut on poly-L-lysine-coated slides for histopathological examination. After deparaffinizing, rehydrating and washing in PBS, the sections were respectively incubated with 5% bovine serum albumin (BSA) in 37 °C oven for 0.5 h. Then, the sections were incubated with mouse ant-CD31 (1:400, Abcam, Cambridge, UK), rabbit ant-PDGFR-β (1:400, Abcam), rabbit ant-p120 (1:400, Abcam), rabbit ant-claudin-5 (1:100, Santa Cruz, CA, USA), rabbit ant-GRP78 (1:400, Abcam) and rabbit ant-CHOP (1:100, Santa Cruz) as primary antibody in 4 °C overnight. The sham control group was operated by using PBS, but not primary antibody. After triple washing in PBS at room temperature, the sections were once again incubated with goat anti-rabbit Alexa Fluor 488 (1:1000, Abcam) and donkey anti-mouse TR (1:1000, Abcam) as secondary antibody for 1 h. For cell immunostaining, the HBMEC subjected to the indicated treatments, were plated on slips. After triple washing in PBS, the slips were fixed in 4% paraformaldehyde for 0.5 h, and blocked with 5% BSA in 37 °C oven for 0.5 h. Then, the slips were incubated with rabbit ant-β-catenin (1:400, Abcam) as primary antibody in 4 °C overnight, following by incubation with goat anti-rabbit Alexa Fluor 488 (1:1000, Abcam) as secondary antibody for 1 h. The nuclei were stained with DAPI for 5 min at room temperature. Images was observed by a confocal microscopy. A negative (no antibody) control was included.

### Western blot analysis

24 rats were euthanized with pentobarbital sodium overdose, and transcardially perfused with 0.9% NaCl 1d after injury. Total proteins from the spinal cord tissues near the lesion (0.5 cm length) and HBMEC were purified using protein extraction reagents. The equivalent of 70 μg of protein was separated on 11.5% gel and transferred onto a PVDF membrane (Bio-Rad Laboratories). The membranes were blocked with 5% skim milk-TBST for 1.5 h at room temperature. Then, the membranes were incubated with primary antibodies as follows: rabbit ant-PDGFR-β (1:1000, Abcam), rabbit ant-p120 (1:1000, Abcam), rabbit ant-β-caternin (1:1000, Abcam), rabbit ant-Occludin (1:1000, Cell Signal Technology), rabbit ant-claudin-5 (1:300; Santa Cruz), mouse ant-ATF-6 (1:1000, Abcam), rabbit ant-PDI (1:1000, Abcam), rabbit ant-GRP78 (1:1000, Abcam), rabbit ant-CHOP (1:300; Santa Cruz), and rabbit ant-caspase 12 (1:1000, Abcam) as primary antibody in 4 °C overnight. After triple washing in TBST, the membranes were incubated with goat anti-rabbit or goat ant-mouse secondary antibody for 1 h. Enhanced chemiluminescence (ECL) kit was used to detect the targeted bands in the membranes. Band intensity was quantified using Image Lab 3.0 software (Bio-Rad Laboratories). Experiments were repeated three times.

### Cell culture

Primary Human Brain Microvascular Endothelial Cells (HBMEC) were obtained from ScienCell Research Laboratories (ScienCell Research Laboratories, San Diego, CA, USA). HBMEC were cultured in Endothelial Cell Medium (ECM), and maintained at 37 °C in a humidified atmosphere containing 5% CO_2_. For a hyperglycemic condition, cells were cultured in ECM with a supplemental glucose (final glucose concentration is 20 mg/mL) for 12 h. H_2_O_2_ (100 μmol/L, Sigma) was used to mimic the SCI environment. To further assess the effects of inhibiting ER stress activation on HG-induced injury, cells were treated with 4-PBA (1 mM) for 2 h with co-stimulation of H_2_O_2_. Then cells were analyzed using immunofluorescence and western blot. All experiments were performed in triplicate.

### Statistical Analysis

Statistical analyses were performed using the SPSS20 statistical software. All data are presented as the means ± SEM. Statistical significance between two experimental groups were analyzed using Student’s t-test. When more than two groups were compared, one-way ANOVA and Dunnett’s *post hoc* test were used. The BBB locomotor scores and the inclined plane test scores were analyzed using Two-way ANOVA followed by Bonferroni *post-hoc* comparison test. P < 0.05 was considered as statistical significance.

## Electronic supplementary material

Supplimentary Figures
