# Author Correction: Inhibition of Endoplasmic Reticulum Stress Preserves the Integrity of Blood-Spinal Cord Barrier in Diabetic Rats Subjected to Spinal Cord Injury

**DOI:** 10.1038/s41598-022-06845-w

**Published:** 2022-02-10

**Authors:** Zili He, Shuang Zou, Jiayu Yin, Zhengzheng Gao, Yanlong Liu, Yanqing Wu, Huacheng He, Yulong Zhou, Qingqing Wang, Jiawei Li, Fenzan Wu, Hua-Zi Xu, Xiaofeng Jia, Jian Xiao

**Affiliations:** 1 Molecular Pharmacology Research Center, School of Pharmaceutical Sciences, Wenzhou Medical University, Wenzhou, 325035 Zhejiang China; 2 Department of Orthopaedics Surgery, The Second Affiliated Hospital and Yuying Children’s Hospital of Wenzhou Medical University, Wenzhou, 325035 Zhejiang China; 3 The Institute of Life Sciences, Wenzhou University, Wenzhou, 325035 China; 4 Department of Neurosurgery, Affiliated Cixi People’s Hospital, Wenzhou Medical University, Ningbo, 315300 China; 5 Department of Neurosurgery, Orthopaedics, Anatomy Neurobiology, University of Maryland School of Medicine, Baltimore, MD 21201 USA; 6 Department of Biomedical Engineering, Anesthesiology & Critical Care Medicine, The Johns Hopkins University School of Medicine, Baltimore, MD 21205 USA

Correction to: *Scientific Reports* 10.1038/s41598-017-08052-4, published online 09 August 2017

This Article contains an error in Figure 2, where the PDGFR-b bands in panel C are a duplication of P120 bands in Figure 3, panel A. The correct Figure 2 and accompanying legend appear below.

**Figure 2:**
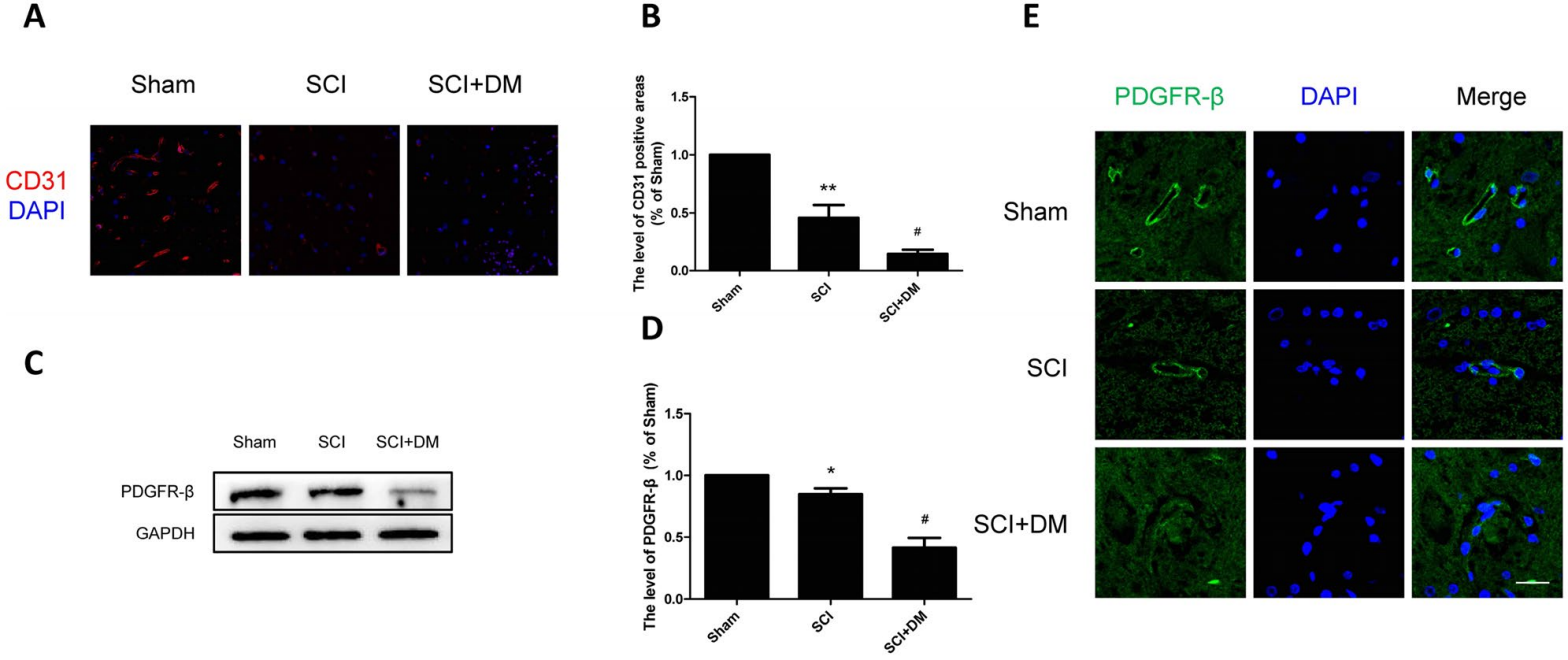
DM aggravates microvessels loss and decreases pericyte coverage after SCI. (**A**,**B**) Immunofluorescence staining of CD31 and quantification of the level of CD31 positive areas in each group, n = 4. Scale bar = 50 μm. (**C**,**D**) Western blot and quantification of PDGFR-β in each group, n = 6. (**E**) Immunofluorescence staining of PDGFR-β in each group, n = 4. Scale bar = 10 μm. *p < 0.05, **p < 0.01 vs sham group, ^#^p < 0.05 vs SCI group.

