# Supplementary material for: Inhibition of Endoplasmic Reticulum Stress Preserves the Integrity of Blood-Spinal Cord Barrier in Diabetic Rats Subjected to Spinal Cord Injury
Source: Sci Rep. 2017 Aug 9;7:7661. doi: 10.1038/s41598-017-08052-4 (PMC5550423; doi:10.1038/s41598-017-08052-4)
Supplement: Supplementary file 1 — Supplimentary Figures [file 41598_2017_8052_MOESM1_ESM.pdf]

Inhibition of Endoplasmic Reticulum Stress Preserves the Integrity of Blood-Spinal Cord Barrier in Diabetic Rats Subjected to Spinal Cord Injury.

Zili He [1,2#], Shuang Zhou [1#], Jiayu Yin [1], Zhengzheng Gao [1], Yanlong Liu [1], Yanqing Wu [3], Huacheng He [3], Yulong Zhou [2], Qingqing Wang [1,2], Jiawei Li [1,2], Fenzan Wu [4], Hua-Zi Xu [2], Xiaofeng Jia [5,6*], Jian Xiao [1,2*]

1 Molecular Pharmacology Research Center, School of Pharmaceutical Sciences, Wenzhou Medical University, Wenzhou, Zhejiang, 325035 China
2 Department of Orthopaedics, The Second Affiliated Hospital and Yuying Children's Hospital of Wenzhou Medical University, Wenzhou, Zhejiang, 325035 China
3 The Institute of Life Sciences, Wenzhou University, Wenzhou 325035, China
4 Department of Neurosurgery, Affiliated Cixi People's Hospital, Wenzhou Medical University, Ningbo, 315300, China
5 Department of Neurosurgery, Orthopaedics, University of Maryland School of Medicine, Baltimore, MD 21201, USA
6 Department of Biomedical Engineering, The Johns Hopkins University School of Medicine, Baltimore, MD 21205, USA

[#] These authors have contributed equally to this work.

Correspondence
Jian Xiao, School of Pharmaceutical Sciences, Wenzhou Medical University, Wenzhou, Zhejiang 325035, PR China.
Tel.:+86-577-85773087
Fax.: +86-577-85773087
E-mail: xfxj2000@126.com
Xiaofeng Jia, Department of Neurosurgery, Orthopaedics, University of Maryland School of Medicine, Baltimore, MD 21201, USA
Tel.: +1-410-706-5026
E-mail: xjia@som.umaryland.edu

Supplementary data

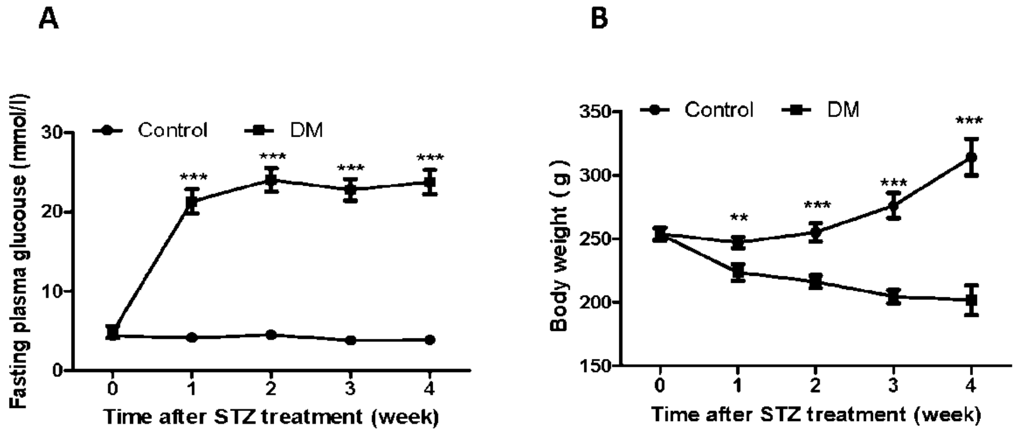

Supplementary Figure. 1. Induction of Type 1 diabetes with STZ. Time course of fasting blood glucose concentration (A) and body weight (B) after streptozotocin (DM) or citrate buffer (control) treatment. Data are presented as mean ± SEM. n = 8, ** p < 0.01, *** p < 0.001 vs control group.

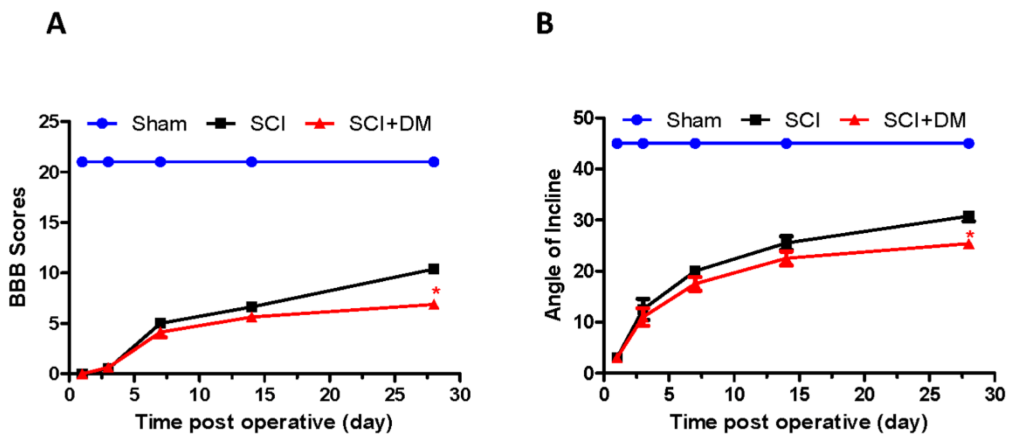

Supplementary Figure. 2. DM aggravates locomotor dysfunction after spinal cord injury (SCI), with decreased BBB scores (A) and inclined plane test scores (B) of the sham, SCI, and SCI + DM groups. Data are presented as mean ± SEM. n = 8, * $p < 0.05$ vs SCI group.

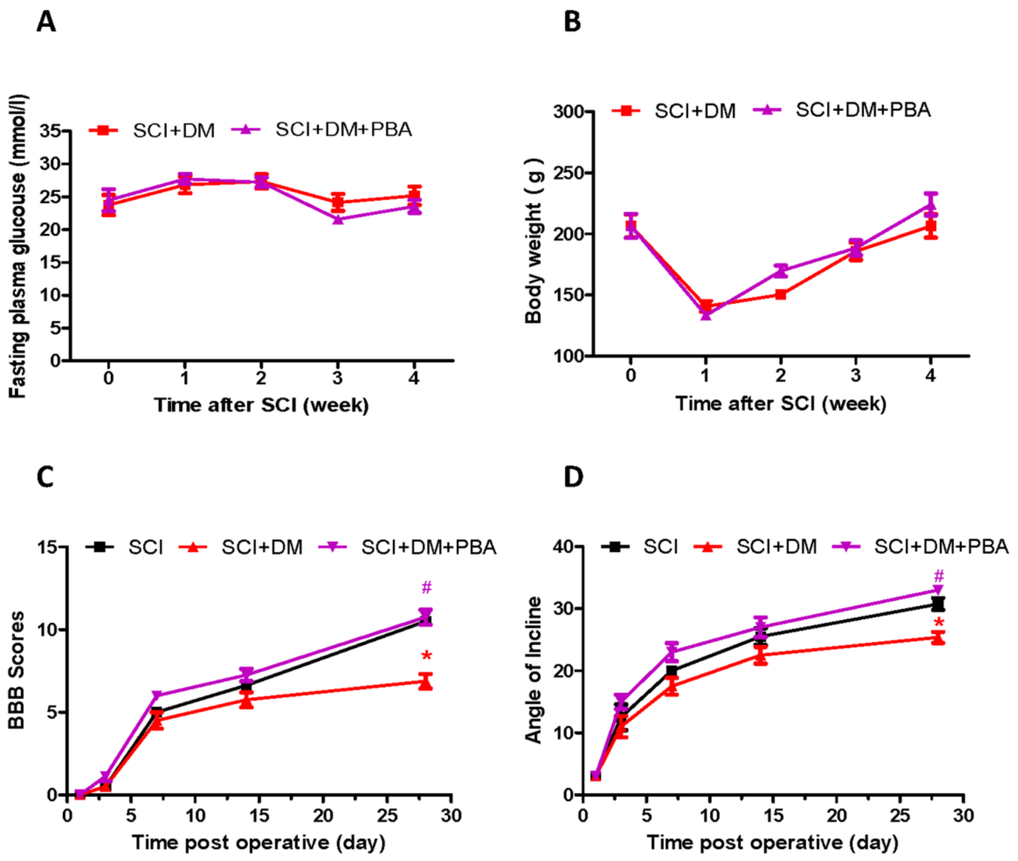

Supplementary Figure. 3. 4-PBA does not change fasting plasma glucose and body weight, but improves locomotor function in diabetic rats after SCI. Time course of fasting blood glucose concentration (A) and body weight (B) of the SCI, SCI + DM, and SCI + DM + PBA groups. The BBB scores (C) and inclined plane test scores (D) of each group. Data are presented as mean ± SEM. n = 8, * $p < 0.05$ vs SCI group, # $p< 0.05$ vs DM+SCI group.

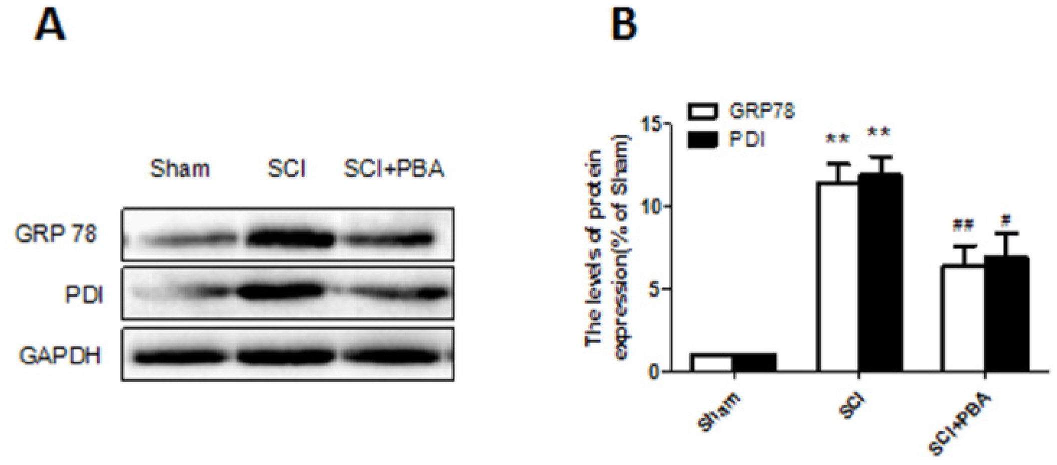

Supplementary figure 4

Supplementary Figure. 4. PBA inhibits the activation of ER stress after SCI. (A, B) Western blot and quantification of GRP78 and PDI in sham, SCI and SCI +PBA group, n = 6. ** $p < 0.01$ vs sham group, # $p < 0.05$, ## $p < 0.01$ vs SCI group.

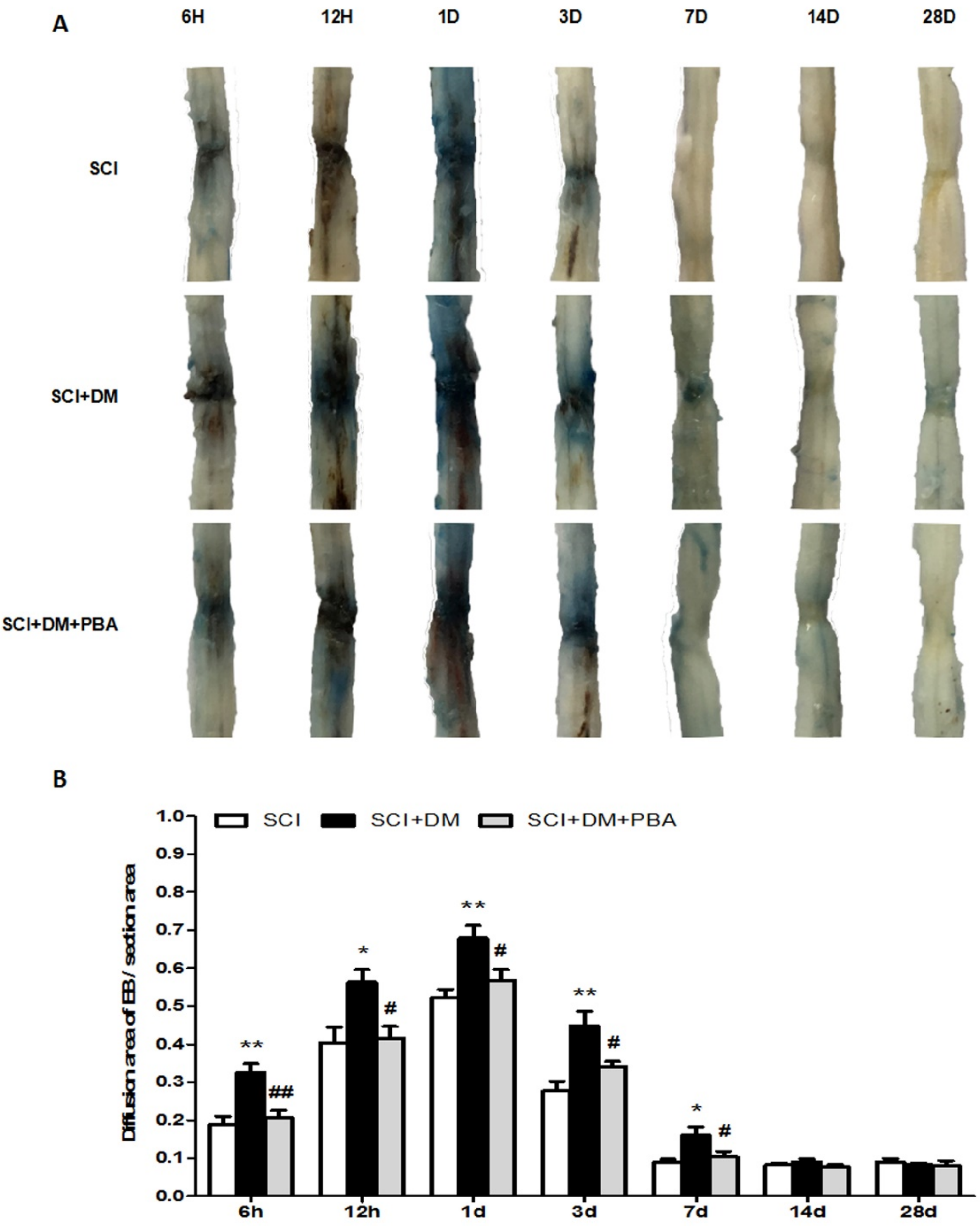

Supplementary Figure 5. Blood–spinal cord barrier permeability after SCI at 6h, 12h, 1d, 3d, 7d, 14d, and 28d after injury, using Evans Blue (EB) dye in SCI, SCI + DM and SCI + DM + PBA group. (A) Representative whole spinal cords and (B) quantification of BSCB permeability data in each group showing EB dye permeabilized into the spinal cord. n = 3 per group, * $p < 0.05$, ** $p < 0.01$ vs sham group, # $p < 0.05$, ## $p < 0.01$ vs SCI group.
